# How does social integration influence breast cancer control among urban African-American women? Results from a cross-sectional survey

**DOI:** 10.1186/1472-6874-8-4

**Published:** 2008-02-06

**Authors:** Ann Carroll Klassen, Carmen Washington

**Affiliations:** 1 Department of Health, Behavior and Society, Johns Hopkins Bloomberg School of Public Health, Baltimore MD, USA; 2 University of Washington School of Social Work and School of Public Health, Seattle WA, USA

## Abstract

**Background:**

Although social integration is a well-established influence on health, less is known about how the specific types of social connection (social roles, social networks, and social support) influence knowledge, attitudes, and practices for specific prevention goals, and how to utilize these influences in interventions with priority populations. This research examined the prevalence of social roles, networks and support among 576 urban African-American women age 45–93 in East Baltimore, Maryland, and the association of these social factors with breast cancer related knowledge, attitudes, and practices.

**Methods:**

Using data from 1997–1998 in-home interviews, we developed indices of six possible social roles, social networks of family, neighborhood and church, and instrumental and emotional social support. In multivariate models adjusting for age, education, and medical care, we examined the association of each social influence on breast cancer knowledge, attitudes, screening recency and intention, and treatment preferences.

**Results:**

We found substantial variation in social integration among these women, with social integration positively associated with overall health and well-being. Social roles and networks were positively associated with screening knowledge, and emotional support and church networks were positively associated with attitudes conducive to early detection and treatment. In regard to screening behaviors, family networks were associated with both screening recency and intention. Women with greater church networks and emotional support held more conservative attitudes towards lumpectomy, reconstruction, and clinical trials.

**Conclusion:**

Overall, social integration is a positive influence on breast cancer control and should be utilized where possible in interventions, including identifying surrogate mechanisms for support for subgroups without existing social resources.

## Background

There has been substantial success in reducing the societal level of burden from breast cancer in the United States over the past several decades. Although breast cancer incidence continues to grow, survival rates have also improved, an accomplishment attributed to multiple factors, including increased early detection, and improved treatment results among women diagnosed [1,2]. Despite improved survival rates among all groups, rates for African-American women still lag behind white women, due in part to less favorable disease characteristics, but also to lower rates of early detection and later stage at diagnosis [2-5].

In order to reduce race-based breast cancer disparities, increasing mammography uptake and consistent use has been an important goal. Structural barriers have been addressed through programs to increase availability and reduce cost [6]. A complementary focus requires understanding and addressing social and psychological barriers to the adoption of healthful cancer-related attitudes and practices among at-risk populations [3,4,7].

The importance of social integration to health behaviors and health has long been established [8-11]. Theory defines at least three conceptually different aspects of a person's social sphere, and the degree to which they are integrated or embedded into those around them. Social roles are obligations, which establish social control by demanding normative behaviors, but also offer social rewards. Social networks represent affiliations, offering knowledge, assistance and connectedness to others, with loose affiliations to multiple networks seen as most healthful. Social support, a product rather than a structure, represents assistance a person can count on as a buffer or aid in problem solving. As many as four subtypes have been conceptualized: emotional, informational, appraisal, and instrumental.

The value of social integration in promoting preventive health behaviors has long been of interest to health researchers, but methodological issues remain. Often, relationships between social resources and specific behavioral outcomes are examined, with less effort made to elucidate possible pathways. Additionally, overlapping concepts are often blended; for example, social support is often measured by networks [12]. Thus, networks are assumed to be supportive when actually they can be burdensome, especially in communities or cultures with substantial rates of poverty, poor physical and mental health, and other social problems.

For breast cancer-related behaviors such as mammography, it is important to understand the possible pathways through which consistent screening is achieved, and the ways in which each of these social resources might foster or impede mammography maintenance. Theories such as the Health Belief Model [13] and Precaution Adoption Model [14] propose that preventive health behaviors are adopted under certain circumstances when perceived benefits outweigh drawbacks. Accurate knowledge of the appropriate timing of screening initiation and periodicity are important. Attitudes consistent with cancer control behaviors include belief that early detection is beneficial, i.e., that it leads to a better outcome than later detection. Detection of cancer must be seen an acceptable outcome of screening uptake, and the subsequent treatment, while unpleasant, must be viewed as preferable to ignoring the disease. Some sense of both initial and ongoing risk for the disease, and therefore need for ongoing vigilance, must be perceived.

Social factors could influence many of these pathways. Prevention knowledge comes in part through mediated and interpersonal messages from health professionals; however, friends, coworkers, neighbors and family members are important to both introduce and reinforce health messages [15]. Our attitudes towards illnesses such as cancer are strongly influenced by our social network members. Our drive to prevent illness comes from others; we maintain health to meet the demands of our social roles. Preventive health actions themselves can be facilitated by help we receive from those in our networks, but can also be restricted by obligations to others that drain time, resources, or stamina.

The substantial existing literature reveals conflicting results from studies examining social resources and preventive behaviors. Antonucci [16] found that social roles, measured by marital and work status, had little impact on cancer detection practices, although marriage was associated with decreased screening among women. Kelsey [17] found among both white and black female blue collar workers that social networks (combining marriage, family, friends, church and organizational affiliations) were predictive of primary but not secondary cancer prevention behaviors, and that co-worker-based social support, measured by both social interactions and health-related discussions, positively predicted physical activity and Pap test receipt. Suarez [18] reported that social networks increased knowledge of mammography guidelines among Mexican and Cuban American women, but not Central American or Puerto Rican women.

Two studies focusing exclusively on African-American women report mixed effects for networks, perhaps due to differing measurement approaches. Kang [12] found a positive association between social network index scores and routine mammography, and Husanini [19] reported that marriage but not church increased breast cancer screening participation.

There is also mixed evidence for a link between social support and breast cancer screening. In contrast to the above-cited findings on social networks, Kang [20] found no association between instrumental or emotional support and mammography. Katapodi [21] also saw no association between mammography and a measuring blending instrumental, emotional, and informational support, in a population of Latina, African-American and white women. However, Farmer found that women reporting a mammogram within the past year had higher scores on a combined index of perceived emotional and functional support [22]. A British study [23] examined both support and networks and found only the number of close friends increased mammography adherence. Finally, one study considered both potential negative and positive effects of social resources among women age 50–79, and found that emotional and informational support were positive predictors of mammography adherence, but that the social role of caregiving was associated with lower likelihood of annual screening [24].

### Goals of This Work

The purpose of this analysis was to clearly define social roles, social networks and social support among low income urban African American women, and explore the relationship between these factors and knowledge, attitudes and practices related to breast cancer screening and treatment. It is important to better understand the factors which prevent high risk populations from maintaining important health behaviors, in order to potentially design interventions targeted to these groups.

This is especially important when intervening in communities where multiple supports are needed to help low resource populations overcome the substantial barriers to adopting multiple health maintenance behaviors on a consistent long term basis. If we better understand how specific social resources function to reinforce health behaviors, this could serve two important purposes. For subgroups with resources, we can more fully make use of existing supportive mechanisms in program design and message. In addition, we can purposefully create alternative supportive mechanisms for subgroups lacking the naturally occurring versions.

## Methods

### Population

Data used in these analyses come from a multi-year National Cancer Institute-funded study of breast cancer screening among African American women in Baltimore, Maryland, a large city on the East Coast of the United States. Methods and related findings have been previously published, and will be briefly described here [25]. With the original goal of evaluating the impact of a no-cost screening intervention within communities at risk for poor screening, we recruited women from 10 continguous zipcodes in East Baltimore who had attended a no-cost mammography program and a matched sample of participant-nominated friends and neighbors not attending the program. A 90-minute, in-home audiotaped interview was conducted by African-American female interviewers. During 1997 and 1998, interviews were completed with 576 women between the ages of 45 and 93 (85% response rate). Participants provided written informed consent, and received $25 for participation. The study was approved by the Johns Hopkins Medical Institutions institutional review board.

The original case-control design was chosen to evaluate the impact of the screening program. In addition, comparison of respondents to Census-based sociodemographic characteristics of their neighborhoods supports analysis of the total group as a representative population of low and moderate income urban African-American women, for questions not specifically related to the no-cost program [26-28].

### Measures

#### Social Resource Measures

We combined questionnaire measures to create indices operationalizing three distinct types of social resources: social roles, social networks, and social support. We further refined these three areas by constructing separate indices for social network subdomains of family, neighborhood and church, and emotional and instrumental subdomains within social support, for a total of six separate indices.

We counted questionnaire responses on six possible social roles to create a social roles index (range 0–6): whether the respondent reported she was currently a spouse, parent to a living child, full or part-time worker, household member (ie, living with at least one other person), paid or unpaid caregiver to a child under age 18 or disabled adult, and community leader (serving on board or committees, or organizing events or activities).

The family social network index ranged from 0–5, summing items on spouse, number of living children (1–2 = 1,3–4 = 2, 5–13 = 3), and close relatives living nearby. Neighborhood social network index scores ranged from 0–6, scored on homeownership, neighborhood organization participation, residential tenure (5–19 years = 1, 20+ years = 2), strong feelings of belonging to her neighborhood, and knowing neighbors very well. Church social network (0–6) measured attendance (1–4/month = 1, 5+ = 2), and closeness to both leaders and fellow members (somewhat close = 1, very close = 2).

Emotional social support (0–4) summed whether or not respondents reported socializing with neighbors, receiving support from family and close friends, and having someone who is concerned about their health and taking care of themselves. Instrumental support (0–4) summed financial help from family, lending help from neighbors, having sufficient current help, and having an expected source of additional help if needed. Full wording of each questionnaire item used is given in additional file 1.

#### Other Measures

Sociodemographic measures used in these analyses included self-reported age, years of formal schooling, and annual household income. Health-related covariates included current depressive symptoms, measured by a brief version of the Center for Epidemiological Studies depression inventory (CES-D) [29], current smoking status, current self-assessed health, and any of a list of 15 chronic illnesses.

As outcomes, we examined 11 measures of breast cancer-related knowledge, attitudes, screening practices and treatment-related preferences. Two measures of mammography knowledge include whether or not respondents correctly described mammography as an X-ray of the breast, and whether they accepted the need for continuous screening by somewhat or strongly disagreeing with the statement "After two or three negative mammograms, it is not necessary to have any more." Attitudes conducive to early detection and treatment of breast cancer were measured by agreement or disagreement with the following statements "If I had cancer, I would rather not know about it.", "Cancer is the worst disease I can imagine having." and "Breast cancer treatments are worse than the disease.", as well as a yes/no question, "Do you feel there is such a thing as being cured of breast cancer?" Mammography practices were measured by whether the respondent reported a mammogram within the past year, and whether she intended to have any mammograms in the future.

To further explore respondents' feelings about breast cancer control, we asked several questions about attitudes towards various aspects of breast cancer treatment. Respondents were asked whether they would choose to have a lumpectomy or a mastectomy, if given the choice, whether they would have breast reconstruction surgery if they had a breast removed, and whether they would ever be willing to have a research or experimental treatment for an illness, if offered.

### Analysis

We report the frequencies for each item within the indices, as well as the range, mean, and standard deviation for each index (see additional file 1). Table 1 reports Pearson correlation coefficients for pairwise association between indices. In table 2, we describe the distribution of social resources within the respondent population, by reporting average values on each index by sociodemographic covariates, and testing differences between these means with t-tests.

**Table 1:** Pearson Correlations Between Six Indices of Social Resources for Urban African-American Women (n = 576)

	Family Network	Neighborhood Network	Church Network	Emotional Support	Instrumental Support
Social Roles	0.46	0.17	0.02	-0.09	0.10
	P < .001	P < .001	P = .59	P = .03	P = .02
Family Network		-0.01	0.04	-0.07	0.04
		P = .88	P = .28	P = .08	P = .35
Neighborhood Network			0.20	0.09	0.13
			P < .001	P = .02	P = .002
Church Network				0.13	0.09
				P = .002	P = .04
Emotional Support					0.22
					P < .001

**Table 2:** Average Social Factor Score by Psychosocial Characteristics of Urban African-American Women (n = 576)

	Roles		Family		Neighborhood		Church		Instrumental		Emotional	
Age group												
45–60 (n = 275)	3.5	P < .001	3.0	P = .40	2.8	P < .001	3.6	P = .001	2.3	P = .16	2.6	P < .001
61–93 (n = 301)	2.6		2.9		3.5		4.1		2.2		2.8	
Education												
<HS (n = 323)	2.8	P < .001	3.0	P = .11	3.0	P < .001	3.7	P = .002	2.2	P = .90	2.7	P = .50
≥ HS (n = 253)	3.3		2.9		3.5		4.2		2.2		2.7	
Yearly Income												
≤$10000 (n = 252)	2.5	P < .001	2.8	P = .02	2.8	P < .001	3.7	P = .04	2.2	P = .85	2.8	P = .02
>$10000 (n = 324)	3.4		3.0		3.5		4.0		2.2		2.6	
Depressive Sx												
None (n = 283)	3.0	P = .99	2.9	P = .76	3.4	P = .001	4.1	P = .008	2.2	P = .85	2.8	P = .04
Any (n = 293)	3.0		3.0		3.0		3.7		2.2		2.6	
Current Smoker												
Yes (n = 154)	3.0	P = .97	2.8	P = .18	2.8	P < .001	3.2	P < .001	2.3	P = .02	2.6	P = .38
No (n = 422)	3.0		3.0		3.3		4.1		2.2		2.7	
Health Status												
Fair/Poor (n = 254)	2.8	P < .001	2.9	P = .85	2.9	P < .001	3.7	P = .01	2.2	P = .81	2.7	P = .88
Good/Exc(n = 322)	3.2		3.0		3.4		4.1		2.2		2.7	
# of Chronic Cond												
0, 1, 2 (n = 225)	3.3	P < .001	3.0	P = .13	3.1	P = .40	3.8	P = .45	2.2	P = .63	2.6	P = .01
3–8 (n = 351)	2.9		2.9		3.3		4.0		2.2		2.8	

Table 3 explores the relationship between social resources and breast cancer. We report the results of multivariable linear regression models, testing differences in average score for each of the six social resource indices by breast cancer-related knowledge, attitudes, practices, and hypothetical treatment choices. Models for knowledge and attitude measures include covariate adjustment for respondent age and education, and models for screening practices and treatment preferences adjust for age, education, and having a usual source of medical care. These covariates are centered at respondent median age (62 years), education (11 years), and most common response (having care (91%)).

**Table 3:** Average Social Influence Scores by Mammography Knowledge, Attitudes, and Practices

		Social Roles				Social Network Index				Social Support Index			
Knowledge^1^		(Number)		Family		Neighborhood		Church		Emotional		Instrumental	

Mammograms are X-Rays	Yes (40%)	3.1	P = .63	3.1	P = .03	3.4	P = .01	4.1	P = .07	2.7	P = .47	2.2	P = .49
of the breast.	No (60%)	3.0		2.9		3.1		3.8		2.7		2.2	
Mammograms are needed	Yes (80%)	3.1	P = .02	3.0	P = .49	3.2	P = .24	4.0	P = .18	2.7	P = .93	2.2	P = .60
continually.	No (20%)	2.8		2.9		3.1		3.7		2.7		2.2	
Attitudes:^1^													
If I had cancer, I would	Yes (17%)	3.1	P = .76	2.8	P = .36	3.0	P = .20	3.6	P = .09	2.6	P = .07	2.2	P = .82
rather not know about it.	No (83%)	3.1		3.0		3.2		4.0		2.7		2.2	
There is such a thing as being	Yes (74%)	3.1	P = .05	3.0	P = .70	3.2	P = .95	4.0	P = .02	2.7	P = .62	2.3	P = .78
cured of breast cancer.	No (26%)	2.9		2.9		3.2		3.6		2.7		2.2	
Cancer is the worst disease	Yes (61%)	3.1	P = .74	3.0	P = .51	3.2	P = .95	3.9	P = .99	2.7	P = .26	2.2	P = .05
I can imagine having.	No (39%)	3.0		2.9		3.2		3.9		2.7		2.3	
Breast cancer treatments are	Yes (57%)	3.1	P = .16	3.0	P = .84	3.2	P = .84	3.9	P = .59	2.6	P = .005	2.2	P = .47
worse than the disease itself.	No (43%)	3.0		2.9		3.2		4.0		2.8		2.2	
Screening Practices:^2^													
Had a mammogram within	Yes (73%)	3.1	P = .56	3.0	P = .04	3.3	P = .72	4.0	P = .60	2.7	P = .40	2.2	P = .45
the past year	No (27%)	3.0		2.8		3.2		3.9		2.7		2.2	
Intends to have a mammogram	Yes (91%)	3.0	P = .94	3.0	P = .004	3.2	P.30	4.0	P = .91	2.7	P = .52	2.2	P = .01
In the future.	No (9%)	3.0		2.5		3.5		3.9		2.8		2.0	
Hypothetical Choices:^2^													
Would Choose Lumpectomy	Yes (57%)	3.2	P = .66	3.2	P = .64	2.9	P = .32	3.4	P = .007	2.8	P = .88	2.1	P = .88
Over mastectomy	No (43%)	3.3		3.3		2.7		3.8		2.8		2.1	
Would Choose Reconstruction	Yes (35%)	3.2	P = .42	3.2	P = .68	2.7	P = .23	3.3	P = .07	2.8	P = .91	2.1	P = .78
after Mastectomy	No (65%)	3.3		3.3		2.9		3.6		2.8		2.1	
Would have an experimental	Yes (49%)	3.3	P = .19	3.2	P = .04	2.9	P = .20	3.6	P = .09	2.7	P = .09	2.1	P = .12
treatment if offered	No (51%)	3.2		3.3		2.7		3.4		2.9		2.0	

Adjusted for age, education (1), and having a current source of care (2). Centered at mean age (62), education (11), and having care.

## Results

Univariate information is provided on the six indices and their composite elements (see additional file 1). The average number of social roles was three; parent and multi-person household member were the most common roles, and spouse and community leader the least common. In terms of social networks, the family subscale similarly indicates that several children, as well as nearby, closely connected relatives, were common network elements for most women, with a spouse being far less common.

The neighborhood network subscale reveals subjective assessments such as belonging and knowing neighbors, as well as residential tenure and homeownership, were common network elements for many respondents. Actual participation in community organizations and meetings was less common, although not rare. Some degree of church-related networks was typical for most respondents; approximately half were frequent attenders, and felt very close to church leaders and fellow members.

The two types of social support were also relatively common, with perhaps more evidence of emotional than instrumental support. Few women reported receiving financial help from family or sharing small items with neighbors. Despite this, they overwhelmingly felt they had sufficient help currently, as well as help to call on if ill or in need. Emotional support was very common. The majority of women reported socializing, receiving support from both family and friends, and also having at least one person who was concerned about their health and whether they were "taking care of themselves".

Table 1 provides evidence that these six measures tapped into related but not completely overlapping domains of social resources. Social roles were strongly positively correlated with family networks, and weakly with neighborhood networks. A greater number of social roles had a weak positive correlation to a woman's perception of available instrumental help, but was negatively, and weakly correlated with emotional support.

There was no significant correlation between the size of family networks and either of the two non-family networks; however, church and neighborhood network size had a moderate positive correlation. Larger family networks were not associated with greater emotional or instrumental support, but both neighborhood and church networks had weak positive correlation to emotional and instrumental support. These two findings suggest that non-family networks function differently than those within families. Finally, emotional and instrumental support were moderately correlated; suggesting that these two capture overlapping but distinct aspects of support.

Table 2 shows bivariate tests of mean differences in social resource index scores by psychosocial attributes, and provides support of the convergent and discriminant validity of these social resource indices. For example, older women held fewer social roles than younger women. However, they also reported stronger neighborhood and church networks, and felt they had access to greater emotional support than younger women. Greater educational attainment when young was associated in older age with more social roles, and increased neighborhood and church roles. There is evidence that poverty was associated with reduced social resources for these women. Those with lower incomes reported significantly fewer social roles, reduced family, neighborhood and church networks, but did report greater emotional social support.

Overall, social resources were associated with greater mental and physical well-being. Poorer self-assessed health and more than two chronic health conditions were associated with reduced number of social roles, and respondents reporting smoking, depressive symptoms and worse self-rated health had weaker neighborhood and church networks. However, in terms of actual help received, two statistically significant associations suggest that more perceived help was available to those in greater need of help. Smokers reported higher instrumental support, and women with more chronic diseases had greater emotional support.

The results of the multivariate models in Table 3 support the argument that conceptually different types of social resources have significant but different effects on health-related behaviors, knowledge, and past and future actions. Knowledge about mammography appears to have been strongly tied to social structural influences. Controlling for age and education level, women who knew that mammography involved an x-ray of the breast had significantly higher scores on all three types of social networks, with mean differences in family and neighborhood scores more significant that differences in church network scores. Women who knew that continual screening is necessary (by strongly or somewhat disagreeing with the statement "After a woman has two or three negative mammograms, it is not necessary to have any more.") had higher average scores on the social roles index.

The relationships between the four cancer-related attitudes show a different pattern. Social roles were higher among women who agreed that breast cancer can be cured. Additionally, women believing this also had higher scores on the church social network index. Church networks and emotional support were both marginally higher among women who said they would rather know if they had cancer, and instrumental support was marginally higher among women who disagreed with cancer being the "worse disease they can imagine having." Emotional support was significantly higher among women who did not believe that breast cancer treatments are worse than the disease. These patterns suggest that support functions to reduce fear and allow women to face difficult health possibilities.

In terms of actual mammography practices, however, when adjusting for age, education, and access to a medical provider, it is family that appears to have promoted both recency of past screen and future intention to screen. Additionally, instrumental support scores on average were higher among women intending to screen in the future.

Finally, we examined how social resources are associated with choices for hypothetical breast cancer treatment scenarios. Women who said they would choose the more conservative (i.e., more historically common) mastectomy over a lumpectomy were significantly more likely to have higher church networks. No other social resource indices varied by this decision. Similarly, women who would not want to have breast reconstruction after a mastectomy were also more likely to have a trend towards higher church network scores. In terms of considering an experimental treatment (or clinical trial), 51% of respondents said they would not be willing to consider this if offered, and these women showed a trend towards greater family networks and more instrumental support. Taken as a whole, this suggests that women choosing more conservative option in each scenario tended to have higher levels of only certain types of personal ties.

## Conclusion and Discussion

As expected, we see that social integration operates through multiple mechanisms to influence the likelihood that a woman will take up breast cancer screening. From these results, two types of conclusions emerge. The first is to consider how findings, from this specific example in the area of breast cancer, build on our existing understanding of the relationship between social influences and health maintenance. The second is to ask how these findings might be applied, in modifying health education interventions specifically directed towards breast cancer control among low-income, African-American women.

Our findings help to extend prior literature on social embeddedness by examining its prevalence and association to health in a subpopulation who has been understudied in this regard, compared to the majority culture [30]. We see that, even in these disadvantaged communities, robust social roles and social networks are common for women in mid and later life. Consistent with existing literature, number of roles typically declines with aging. However, older women may replace marriage or work ties with fewer but stronger ties with neighbors and church, and derive substantial emotional support through these mechanisms. We also find cross-sectional evidence that role occupancy and the resulting social ties are associated with both mental and physical well-being, and conversely, that poorer social integration is associated with social, psychological, and physical disadvantage – reduced income and education, greater depressive symptoms and tobacco addition, and poorer physical health and chronic illness burden.

This supports earlier evidence that support may be especially important as a buffer for low-resource persons and communities [31]. From a policy perspective, this adds to the evidence that social ties exist even in the most disadvantaged urban communities, and are important resources to reinforce through programs and policies.

Specifically in regard to cancer control, we see that, consistent with previous findings, mammography-related knowledge is enhanced primarily through social structures, and connectedness to the larger society. The depth of a woman's embeddedness in her family, neighborhood, and church increases the likelihood that she can accurately define a mammogram, with stronger influences seen for family and neighborhood networks than for embeddedness in religious organizations. The diversity of her social roles, and number of different social spheres in which she functions, may increase opportunities to learn and accept the cancer control message that continual screening is needed, even after several negative screens have been received.

This would suggest that interventions aimed at increasing knowledge of screening recommendations may be most effective if they disseminate information through family and neighborhood channels, such as "tell-a-friend" or mother-daughter screening campaigns. Church-based educational programs may also have an impact on knowledge.

Furthermore, encouraging low income women to maintain diverse roles as they age may be helpful in maintaining their access to multiple sources of health-related communication and education. However, to reach all women at risk for poor screening knowledge, these results suggest the need to focus new types of educational efforts on older women within communities who are relatively isolated, and do not have these types of social structures through which to receive informal education.

Attitudes conducive to secondary prevention of breast cancer through screening, early detection, and treatment are supported by both roles and networks. Women with a greater number of social roles are more likely to believe that cancer is a curable disease. We might speculate that new norms about cancer as a chronic non-fatal illness are disseminated along informal communication networks, and reinforced by group norms.

Of the three dimensions of social networks, however, only the strength of a woman's church-based network is positively associated with believing in the curability of cancer, and in wanting to know about cancer if she had it. This suggests that social structures which focus on spirituality offer their members unique opportunities to develop positive mindsets towards cancer, and engage in the concept of early detection and treatment. This challenges the view that religiousity leads to fatalistic beliefs and is a negative influence on health-related actions.

Although social support – the emotional and instrumental assistance women reported having – was not related to increased breast cancer-related knowledge, the two types of social support we measured were positively associated with breast cancer-related attitudes. Women with higher levels of emotional support were slightly less likely to want to avoid knowing about a cancer diagnosis, and were much less likely to agree that cancer treatments were worse than the disease itself. This suggests that coping with a hypothetical illness appears less daunting when one has sources of emotional support. Women who disagreed with the idea that cancer is the worse disease imaginable were slightly more likely to report stronger instrumental support, identifying a coping mechanism more related to managing treatment and other practical aspects of a diagnosis.

Most communications about cancer screening do not address the negative aspects of diagnosis and treatment, but instead focus on reducing treatment burden and serious consequences through early detection. However, given the rising prevalence of cancer and cancer survivorship, these results raise an interesting possibility. Improved attitudes about screening may result if persons plan for possible diagnosis by identifying sources of help. The role of emotional help, as well as the role of spirituality and church networks, appears especially beneficial. Among groups targeted for screening, these results suggest that those who do not attend church could especially benefit from structured interventions which link emotional support to screening and work-up, using discussion groups or lay health advisors which address the affective needs of women in regard to breast cancer.

Past screening behaviors and future intentions to screen are related to only two types of social influences. Women reporting receipt of a recent screen (defined as within the past year) have significantly more extensive family networks. Intention to receive a mammogram in the future is highly associated with a more extensive family network, and also with having greater instrumental social support.

This strong positive association with family is remarkable, given the degree to which these women are called on by relatives and children for help. However, these results suggest that this help may be reciprocal, and that children, spouses and nearby relatives in some way may facilitate the receipt of preventive screening. These family members may offer actual assistance, such as childcare or transportation, or reinforcement, such as reminders, discussions and encouragement. To make the best use of this family-based effect, programs might develop additional focus on family-related screening and outreach.

Conversely, women without family networks should be considered at additional risk for poorly sustained screening patterns, and the identification of alternative sources of reinforcing partnerships for these women should be explored. In terms of future intentions to screen, those without strong family networks should be encouraged to identify other important social network members, who could be incorporated into planning for screening reminders, transportation and assistance, or reinforcement. Using known social network members, such as family members, is a specific form of the "lay health advisor model" which has been used successfully in health promotion [32]

The complex influences which are uniquely conferred by intensive involvement with spiritually-based social networks are raised by the final set of findings, regarding hypothetical choices for breast cancer treatment. Although religious involvement, through a strong church social network, is associated with knowledge, attitudes, and practices consistent with early detection and treatment, the final set of findings raise concerns for respondents' openness towards newer treatment options if diagnosed with cancer. After controlling on age, education level, and having a regular source of medical care, three newer treatment choices – lumpectomy, reconstruction, and clinical trials – were all less acceptable to women with church involvement and emotional social support.

It is important to respect the role of personal preference in each of these choices. However, these patterns, when considered together with attitudes showing less overall fear of a cancer diagnosis, raise the possibility that these women might benefit from guided decision-making during cancer treatment and recovery. Church-based interventions could build on positive attitudes towards illness held by the most religiously involved women, by utilizing spiritual perspectives to encourage consideration of all possible technologies and treatments.

There are several limitations to consider when interpreting these findings. We did not have indices of either informational or appraisal social support, such as asking directly about persons with whom you share information, or discuss major decisions. Therefore, we cannot compare these distinct elements of social support to the existing literature. However, the four domains of social support often overlap, which limits the usefulness of comparing all four within the same population [10]. In addition, these data reflect a single geographic area and timepoint, and should be considered together with findings from other populations. Although the data were collected in 1997–1998, our findings that 73% of respondents report a mammogram within the past year is comparable to national mammography rates for African-American women from more recent studies. This reflects the fact that mammography rates among African-American women in the US increased steadily in the 1980's and 1990's, but have leveled off since the late 1990's [2].

As is always the case with cross-sectional reports, we can only speculate about the directionality of these associations. We know that the acquisition of social resources such as education and social relationships such as parenting pre-date recent mammography behaviors. Therefore we can use this causal time order to speculate that where associations are seen, these social influences help form attitudes and behaviors, rather than supposing that breast cancer-specific actions and opinions change a woman's family or work status. However, it is possible that additional factors, such as personality traits, enable women to both maintain relationships and adopt health attitudes and behaviors.

Among these women living in low-resource communities, who represent important targets for continued breast cancer control efforts, this work provides no suggestion that their multiple social roles are a negative influence on their health. Although they are heavily involved in helping their families, churches, and communities, these social relationships do not appear to deter their ability to learn about, accept, and participate in breast cancer screening. Therefore, cancer control programs should make use of existing social resources within communities for women who have them, and creatively work to identify surrogate sources for screening-specific social mechanisms for those who do not.

## Competing interests

The author(s) declare that they have no competing interests.

## Authors' contributions

ACK obtained funding for, designed and conducted the survey, conducted the multivariate analyses, and wrote the manuscript. CW conducted the review of the literature, conducted the exploratory and descriptive analyses, and assisted in the interpretation of the findings. All authors read and approved the final manuscript.

## Pre-publication history

The pre-publication history for this paper can be accessed here:



## Supplementary Material

Additional file 1 Social Roles, Networks, and Support Indices and Index Component Items for Urban African-American Women (n = 576). This table provides exact wording of questionnaire items used to construct indices, scoring for indices, and frequencies for each index item. Click here for file
