# Supplementary material for: How does social integration influence breast cancer control among urban African-American women? Results from a cross-sectional survey
Source: BMC Womens Health. 2008 Feb 6;8:4. doi: 10.1186/1472-6874-8-4 (PMC2262880; doi:10.1186/1472-6874-8-4)
Supplement: Additional file 1 — Social Roles, Networks, and Support Indices and Index Component Items for Urban African-American Women (n = 576). This table provides exact wording of questionnaire items used to construct indices, scoring for indices, and frequencies for each index item. [file 1472-6874-8-4-S1.doc]

| **Social Roles, Networks, and Support Indices and Index Component Items for Urban African-American Women (n=576)** | | |
| --- | --- | --- |
| Index | Wording of Questionnaire Item(s) Used - (Responses coded 1 for index indicated in  **bold**) | % Yes |
| Social Roles | Range 0-6 Mean 3.0 Std Dev 1.3 |  |
| 1) Spouse | Are you currently **married**, widowed, divorced, or have you never been married? | 27% |
| 2) Parent to Living Children | Have you had any children? How many children have you had? Are all of these children still living? (if no) How many have passed away? | 87% |
| 3) Multi-person Household Member | How many people, including yourself, live here in this house with you? | 74% |
| 4) Worker | Are you working now? | 41% |
| 5) Caregiver to Child/Disabled Adult | Do you care for, or help look after any young children on a regular basis? Is there anyone who is an adult, but cannot take care of themselves, who you help care for? | 49% |
| 6) Leadership Position in Community | In the past year, have you served on a board, committee, or worked as a leader or organizer of any community event or activity? | 25% |
| Social Networks |  |  |
| Family Subscale | Index Range: 0-5 Mean: 2.9 Std Dev: 1.1 |  |
| 1) Spouse | See above | 27% |
| 2) Close Relatives Nearby | Do you have relatives who live in Baltimore, but do not live in this house with you? Are you close to some of these relatives, that is, do you see some of them or talk to them on the phone at least once a month? | 95% |
| 3) # of Living Children | See Above |  |
|  | 1-2 =1 | 27% |
|  | 3-4 =2 | 33% |
|  | 5-13 =3 | 27% |
| Community Subscale | Index Range: 0-6 Mean 3.2 Std Dev 1.6 |  |
| 1) Homeowner | Do you own this house/apartment, does someone in your family own it, or do you rent it? | 53% |
| 2) Residential Tenure | How long have you lived in this house/apartment? |  |
|  | 5-19 years = 1 | 39% |
|  | 20 + years =2 | 40% |
| 3) Subjective Sense of Belonging | I feel very much that I belong here in this community (**strongly agree, somewhat agree**, somewhat disagree, strongly disagree) | 64% |
| 4) Knows neighbors very well | How well do you know your neighbors, that is, the people who live in the houses or apartments next door to you? Would you say you know them **very well**, only somewhat, or not at all? | 58% |
| 5) Participates in organizations | In the past year, have you attended any meetings or gotten involved with any community issues or problems? | 43% |
| Church Subscale | Index Range 0-6 Mean 3.9 Std Dev 1.9 |  |
| 1) Attendance | Do you belong to any organized religious group, such as a church, mosque, kingdom hall or temple? (If No): Do you ever go to religious services any place? (For Either): How often do you go to this place, either for services, or some other reason, like meetings or activities? |  |
|  | Monthly-Weekly =1 | 39% |
|  | More than Weekly =2 | 41% |
| 2) Closeness: Leaders | How close do you feel to the person or people who are the leaders of this group, such as the pastors or priests? (Very, somewhat, not very, not at all) |  |
|  | Somewhat close =1 | 31% |
|  | Very close =2 | 53% |
| 3) Closeness: Members | How close do you feel to the other members of this religious group? (as above) |  |
|  | Somewhat close =1 | 39% |
|  | Very close =2 | 47% |
| Social Support |  |  |
| Instrumental Subscale | Index Range 0-4 Mean 2.2 Std Dev 0.7 |  |
| 1) Help: family | Do you receive any regular cash income from your children? | 12% |
| 2) Help: neighbors | How often do you and your neighbors lend each other something, like some food, something to use around the house, or even a small amount of money? (E**very day, at least once a week, every few weeks**, less than once a month, rarely/never) | 27% |
| 3) Enough Help: Now | In general, if you needed someone to help you out, such as giving you a ride to the doctors or bringing you a meal, do you feel you have enough people to help you out, or do you wish you had more? | 90% |
| 4) Enough Help: Future | If you found out you had an illness such as breast cancer and became too sick to take care of yourself as well as you do now, would you have someone you could ask for help? | 93% |
| Emotional Subscale | Index Range 0-4 Mean 2.7 Std Dev 0.8 |  |
| 1) Socializes with neighbors | How often do you get together with anyone from your neighborhood for a visit, either in your home or in theirs? This could be for a meal or a visit, for example, to watch a TV program together in the evening. (**Every day, at least once a week, every few weeks,** less than once a month, rarely/never). | 25% |
| 2) Support: Family | In thinking about your family, please listen to these three statements and tell me which describes your situation the best.  **My family usually gives me a lot of support, and only once in a while causes me stress.**  My family causes me stress as often as it gives me support.  My family usually causes me a lot of stress, and only once in a while gives me support. | 77% |
| 3) Support: Friends | Now in thinking about your close friends, please listen again….  **My friends usually give me a lot of support, and only once in a while cause me stress.**  My friends cause me stress as often as they give me support.  My friends usually cause me a lot of stress, and only once in a while give me support. | 87% |
| 4) Health-specific | Is there any one in your life, either a friend, family member, or someone else who is not a doctor or a nurse, but is worried or concerned about your health, and talks to you about taking care of yourself? | 75% |
